# Co-culture of *Bacillus amyloliquefaciens* ACCC11060 and *Trichoderma asperellum* GDFS1009 enhanced pathogen-inhibition and amino acid yield

**DOI:** 10.1186/s12934-018-1004-x

**Published:** 2018-10-03

**Authors:** Qiong Wu, Mi Ni, Kai Dou, Jun Tang, Jianhong Ren, Chuanjin Yu, Jie Chen

**Affiliations:** 1 School of Agriculture and Biology, Shanghai Jiao Tong University, Shanghai, 200240 China; 2 Anhui Province Key Laboratory of Embryo Development and Reproductive Regulation, Anhui Province Key Laboratory of Environmental Hormone and Reproduction, Fuyang Normal University, Fuyang, 236037 Anhui China; 3 Suzhou BioNovoGene Metabolomics Platform, Suzhou, 215000 China

**Keywords:** *Bacillus amyloliquefaciens*, *Trichoderma asperellum*, Liquid co-cultivation, Amino acid, Biocontrol metabolites

## Abstract

**Background:**

*Bacillus* spp. are a genus of biocontrol bacteria widely used for antibiosis, while *Trichoderma* spp. are biocontrol fungi that are abundantly explored. In this study, a liquid co-cultivation of these two organisms was tried firstly.

**Results and discussion:**

Through liquid chromatography-mass spectrometry/mass spectrometry (LC–MS/MS), it was discovered that with an inoculation in the ratio of 1.9:1, the antimicrobial effect of the co-cultured fermentation liquor of *Bacillus amyloliquefaciens* ACCC11060 and *Trichoderma asperellum* GDFS1009 was found to be significantly higher than that of pure-cultivation. A raise in the synthesis of antimicrobial substances contributed to this significant increase. Additionally, a co-culture with the inoculation of the two organisms in the ratio of 1:1 was found to enhance the production of specific amino acids. This technique could be further explored for either a large scale production of amino acids or could serve as a theoretical base for the generation of certain rare amino acids.

**Conclusions:**

This work clearly demonstrated that co-cultivation of *B. amyloliquefaciens* ACCC11060 and *T. asperellum* GDFS1009 could produce more specific biocontrol substances and amino acids.

**Electronic supplementary material:**

The online version of this article (10.1186/s12934-018-1004-x) contains supplementary material, which is available to authorized users.

## Background

*Bacillus* spp. exhibit antagonistic effects against a variety of pathogenic fungi, as well as multifarious pathogenic bacteria [1]. They are reported to induce pathogen inhibition by antibiosis through the secretion of many secondary metabolites [2]. Different *Bacillus* species secrete diverse substances that include surfactins [3], fengycins [4], bacisubin [5] and polyketides [6]. It has been shown that 3-hydroxypropionaldehyde, produced by *Bacillus*, efficiently inhibits the growth of *Alternaria solani*, *Botrytis cinerea*, *Fusarium sambucinum*, and *Pythium sulcatum* [7].

*Trichoderma* spp. are known to exhibit antagonistic effects against at least 18 genera and 29 species of pathogenic fungi. The biocontrol mechanisms of *Trichoderma* spp. primarily include competition and mycoparasitism, followed by the stimulation of plant resistance and immunity [8]. The most significant antagonistic mechanism of *Trichoderma* spp. against pathogens is mycoparasitism. This process is accompanied by the secretion of cell wall-degrading enzymes (CWDEs) such as chitinases, glucanase, and proteases which penetrate the pathogen mycelium, absorbing its nutrients and ultimately dissolving the pathogen [9, 10]. Additionally, *Trichoderma* spp. are reported to induce pathogen inhibition by secreting secondary metabolites such as 6-pentyl-α-pyrone [11, 12].

Although both *Bacillus* and *Trichoderma* spp. are known to display good biological control, their primary mechanisms for biocontrol are not the same. Despite their capabilities to generate antibacterial secondary metabolites, particular emphasis is needed to control the different chemical compounds synthesized by them. Moreover, the *Bacillus* and *Trichoderma* spp. display different preferences with respect to growth rate, morphology and culture conditions. It would, therefore be very convenient to establish a suitable liquid fermentation process to enable the co-culture of the above two organisms in order to improve the resistance to pathogens, along with harvesting novel chemical compounds during cultivation. It has been reported that a potent anticancerous drug paclitaxel can be produced by the yew tree fungal endophyte *Paraconiothyrium*. Interestingly, when co-cultivated with other fungi such as *Alternaria* sp. or *Phomopsis* sp., the titre of this alkaloid was seen to increase [13]. Similarly, co-culture of fungus *Aspergillus nidulans* challenged by *Streptomyces rapamycinicu*s provided the cue for the molecular basis of induction of silent fungal biosynthetic gene clusters [14, 15]. Thus, co-cultivation methods have been developed and widely applied in the research of microbial secondary metabolites [16].

In this study, the liquid co-cultivation of *Bacillus amyloliquefaciens* ACCC11060 and *Trichoderma asperellum* GDFS1009 was attempted for the first time to harvest their individual advantages such as enhanced antibacterial effect or the stimulated generation of biochemicals. Through LC–MS/MS technology, the similarities and the differences among the antibacterial substances in the co-cultured and individually-cultured fermentation liquors were detected.

## Results and discussion

### The morphology of singly-cultured and co-cultured *B. amyloliquefaciens* ACCC11060 and/or *T. asperellum* GDFS1009 fermentation liquors and their effects on controlling *B. cinerea*

*Bacillus* spp. are a type of bio-control bacteria explored for various large-scale applications with the principal mechanism of antibiosis. *Trichoderma* spp. are a similar bio-control fungi widely used for multiple antimicrobial mechanisms including competition and mycoparasitism.

In this study, the liquid co-cultivation of *B. amyloliquefaciens* ACCC11060 and *T. asperellum* GDFS1009 was attempted for the first time. It was observed that 3 days after their respective single-cultivations, the fermentation liquor B of *B. amyloliquefaciens* ACCC11060 appeared creamy white, while the color of fermentation liquor T of *T. asperellum* GDFS1009 was relatively lighter and appeared beige, with a uniform distribution of mycelia with low viscosity. However, when the inoculation ratio of *B. amyloliquefaciens* ACCC11060 and *T. asperellum* GDFS1009 was 1:1, the fermentation liquor BT1 was bright yellow after 3 days of co-cultivation. The mycelia of *T. asperellum* GDFS1009 were dominant, but they were significantly rarefied and the viscosity was high. Further, when the inoculation ratio of *B. amyloliquefaciens* ACCC11060 and *T. asperellum* GDFS1009 was 1.9:1, the fermentation liquor BT2 was creamy white after 3 days of co-cultivation. The *Bacillus* was found to be dominant, with few *T. asperellum* GDFS1009 mycelia with viscosity assembled together (Table 1).

**Table 1:** Forms and inhibition rates of the fermentation solutions from pure-cultivation and co-cultivation of *B. amyloliquefaciens* ACCC11060 and *T. asperellum* GDFS1009

Name	Inoculation quantity		Fermentation form	Inhibition rate (%)
	*B. amyloliquefaciens* ACCC11060 (mL)	*T. asperellum* GDFS1009 (mL)		
B	1	0	Off-white suspension	58.89 ± 1.34
T	0	1	Beige solution with loose mycelium	30.67 ± 0.95
BT1	1	1	Bright yellow solution with high-viscosity mycelia	47.86 ± 0.51
BT2	1.9	0.1	Off-white suspension with a small amount of high-viscosity mycelium	66.86 ± 2.14

**Fig. 1:**
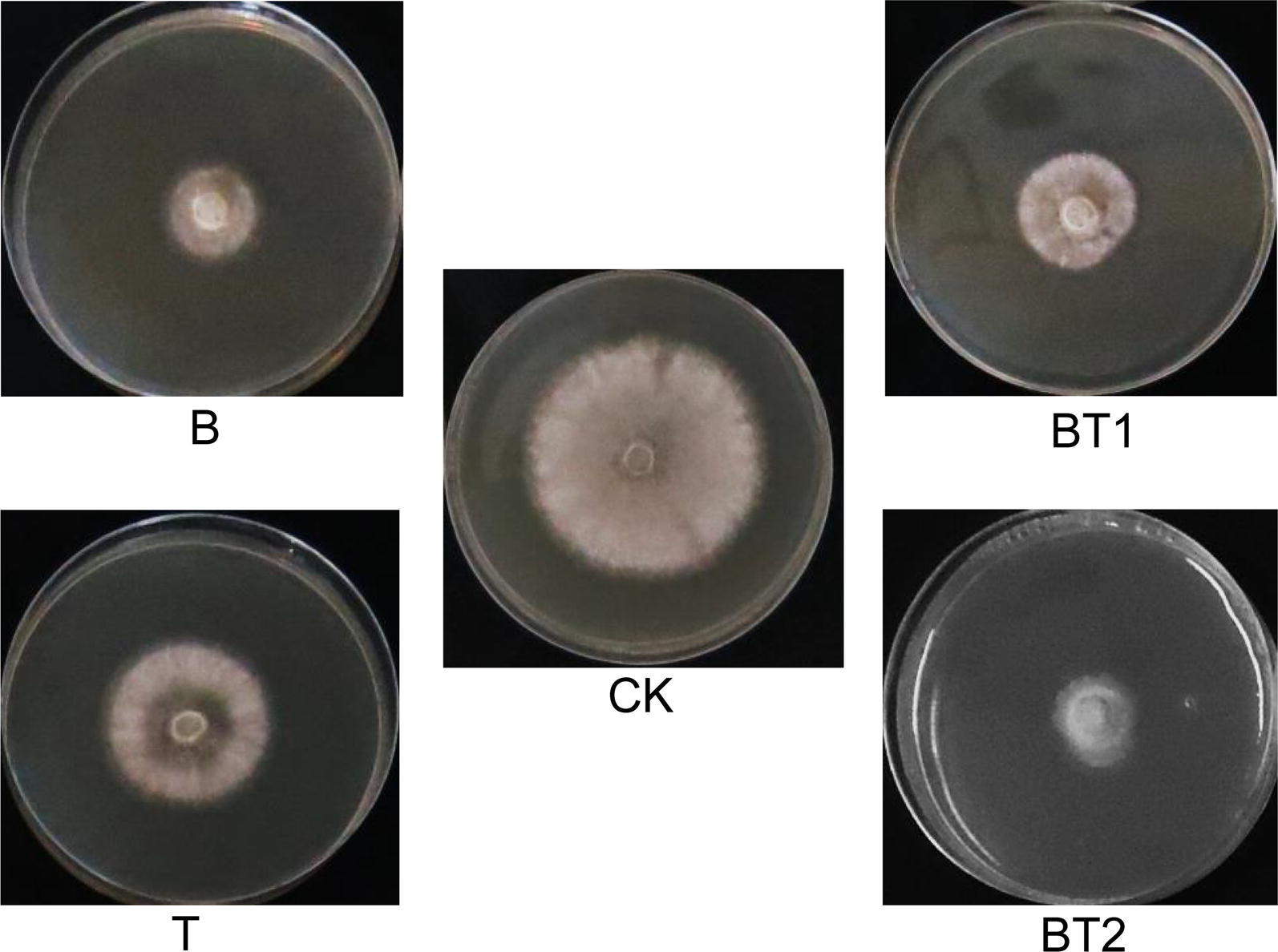
Inhibition effects on *B. cinerea* and changed metabolites of the fermentation solutions

While determining the antibacterial effect of fermentation liquor on *B. cinerea*, it was found that the co-cultured fermentation liquor BT1 exhibited a higher activity (47.86 ± 0.51%) as compared to singly-cultured fermentation liquor T (30.67 ± 0.95%), but was lower than B (58.89 ± 1.34%). Interestingly, the effect of co-cultured fermentation liquor BT2 (66.86 ± 2.14%) was higher than both B and T (Fig. 1 and Table 1).

All the four fermentation liquors-B, T, BT1 and BT2 were subjected to intensive investigations.

### The variance of analysis between fermentation liquor groups of singly-cultured and co-cultured *B. amyloliquefaciens* ACCC11060 and/or *T. asperellum* GDFS1009

**Fig. 2:**
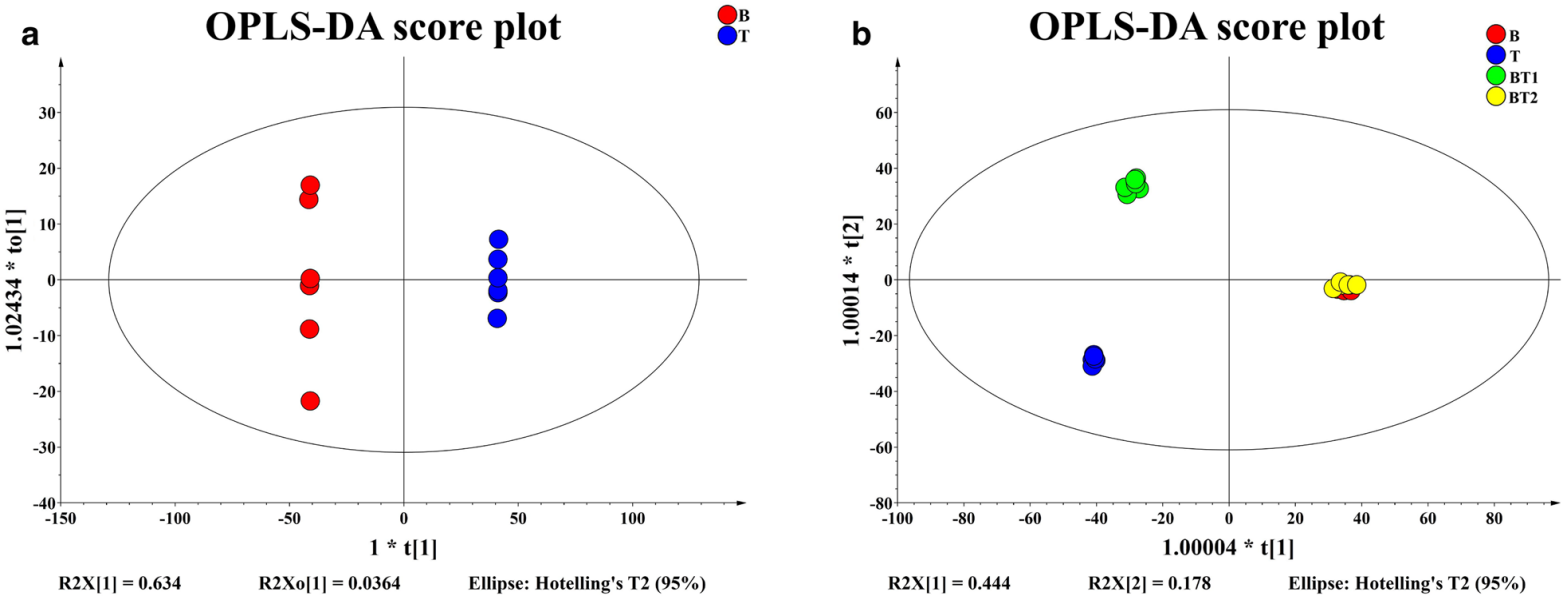
OPLS-DA analysis of the metabolism differences among the fermentation solutions from pure-cultivation and co-cultivation of *B. amyloliquefaciens* ACCC11060 and *T. asperellum* GDFS1009 based on LC–MS. **a** Differences of the pure-cultivations; **b** differences among the pure-cultivations and co-cultivations

After the OPLS-DA analysis of metabolites determined through LC–MS (+) and LC–MS (−), it was found that there were significant differences in the metabolic compositions of B, T and BT1 fermentation liquors. However there were non-significant differences identified between BT2 and B, although both showed certain differences (Fig. 2). This was consistent with PCA analysis (data not shown).

**Fig. 3:**
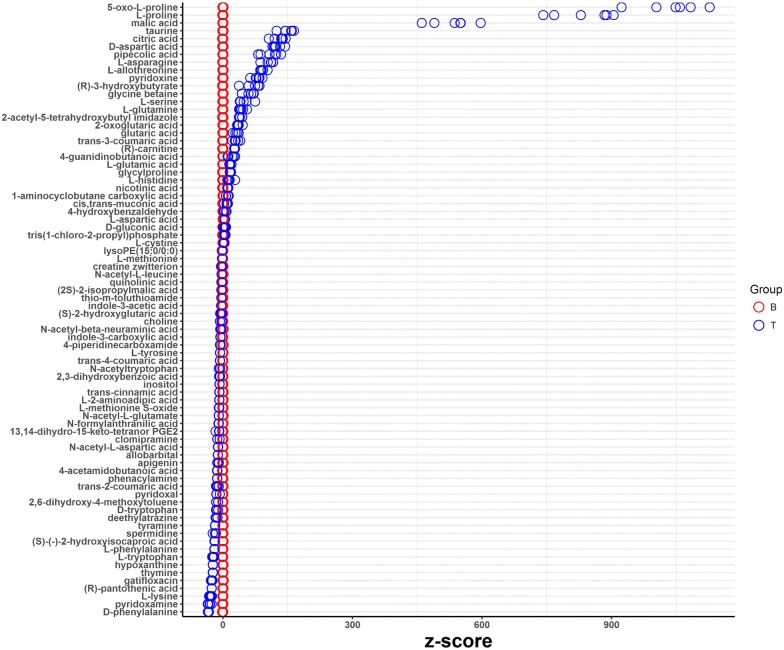
Different metabolites of the fermentation solutions from pure-cultured *B. amyloliquefaciens* ACCC11060 and *T. asperellum* GDFS1009 based on LC–MS/MS analysis

Through LC–MS/MS analysis in the present study, 102 types were successfully annotated among the plethora of various metabolites. When the fermentation liquors T of *T. asperellum* GDFS1009 was compared with B of *B. amyloliquefaciens* ACCC11060, a total of 78 chemical compounds were found to be present with significant differences, with an elevation of 47 and 31 compounds in B and T respectively. The chemical compounds with significant variations included amino acids, organic acids, alcohols, aldehydes, flavonoids and alkaloids (Fig. 3).

### Analysis of differential compounds in singly-cultured and co-cultured *B. amyloliquefaciens* ACCC11060 and/or *T. asperellum* GDFS1009 fermentation liquors through LC–MS/MS

**Fig. 4:**
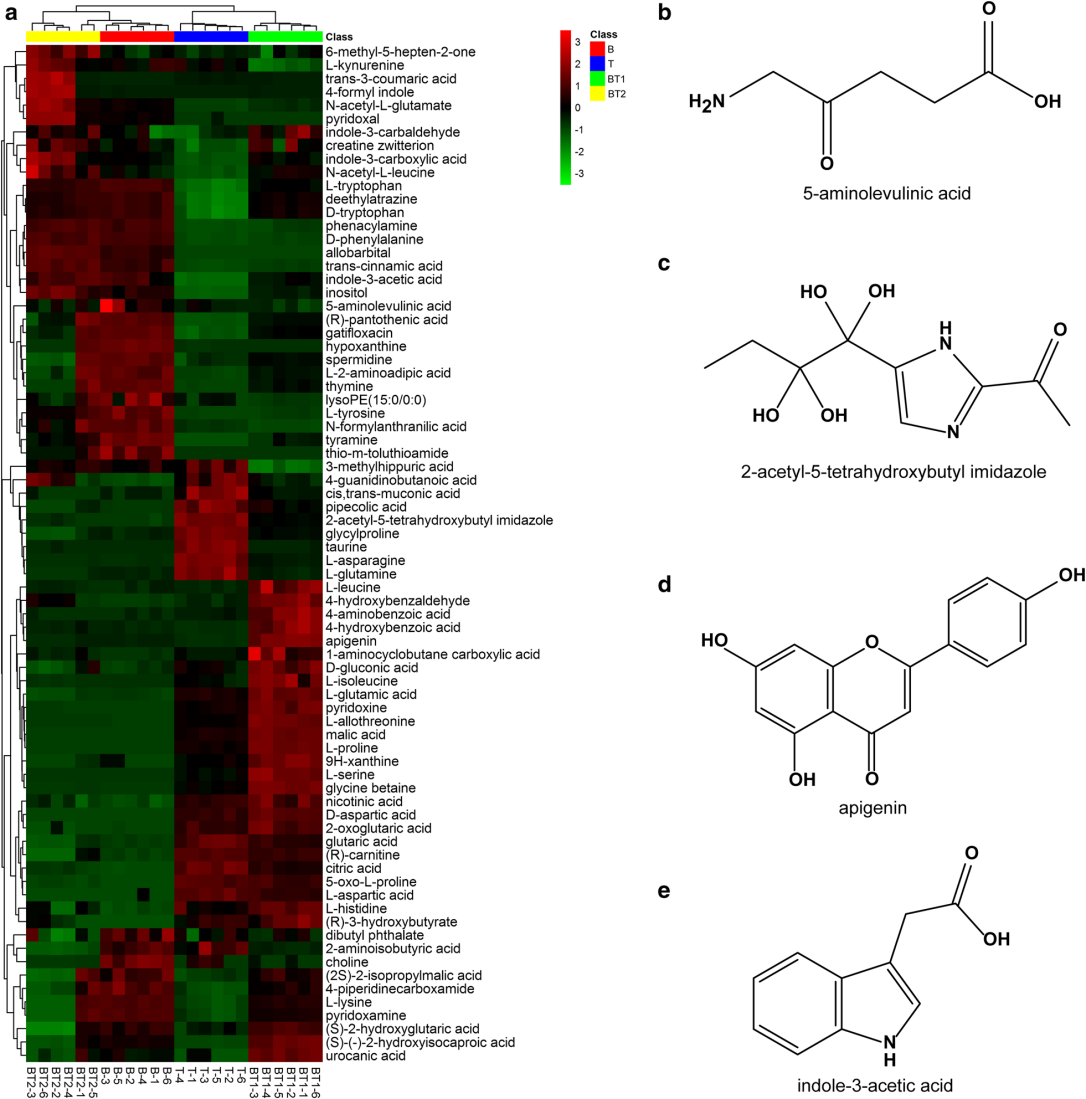
Significantly changed metabolites among the fermentation solutions from pure-cultivation and co-cultivation of *B. amyloliquefaciens* ACCC11060 and *T. asperellum* GDFS1009 based on LC–MS/MS. **a** Global analysis with Heatmap; **b** the metabolite that are only highly productive in B; **c** the metabolite that are only highly productive in T; **d** the metabolite that are only highly productive in BT1; **e** the metabolite that are only highly productive in BT2

Through the LC–MS/MS analysis, 76 compounds were found to exist with significant differences in a comprehensive comparison between B, T, BT1 and BT2 (Additional files 1, 2). A total of 22 compounds were identified with significantly high proportion in B, 14 were found in T, while another 24 and 16 were found in BT1 and BT2 respectively (Fig. 4a).

l-Amino acids have various applications in food and feed biotechnology and they also serve as intermediates in chemical industry [17]. With the positive growth of the amino acid market, the microbial production of amino acids is drawing more attention. Interestingly, in this study, among the compounds with significant variations, it was noticed that amino acids accounted for 23.68%. 3 amino acids were found significantly high in B and included l-lysine, l-tryptophan and l-tyrosine. Similarly, 3 amino acids found significantly high in T included 5-oxo-l-proline, l-asparagine and l-aspartic acid. There were a total of 8 amino acids found in significantly high proportions in BT1 and included d-aspartic acid, l-allothreonine, l-glutamic acid, l-histidine, l-isoleucine, l-leucine, l-proline and l-serine. Lastly, 4 amino acids identified with significant high yields in BT2 included d-phenylalanine, l-kynurenine, *N*-acetyl-l-glutamate and *N*-acetyl-l-leucine (Table 2). Among these, many rare amino acids were found including 5-oxo-l-proline in B, l-allothreonine and l-glutamic acid in BT1, and l-kynurenine, *N*-acetyl-l-glutamate and *N*-acetyl-l-leucine in BT2. Taken together, these data provide an excellent theoretical guidance for large-scale production of specific amino acids in the industry.

**Table 2:** Amino acid with high yield under only one culture condition

Compound	VIP	p-value	Culture condition
d-Tryptophan	1.10083	2.63996E−14	B
l-Lysine	1.27053	3.31824E−05	B
l-Tryptophan	1.34496	3.59313E−18	B
l-Tyrosine	1.11095	1.33916E−12	B
5-Oxo-l-proline	1.02467	5.02938E−19	T
l-Asparagine	1.35007	1.32867E−18	T
l-Aspartic acid	1.00148	5.98419E−13	T
d-Aspartic acid	1.25926	1.18975E−20	BT1
l-Allothreonine	1.11358	3.55869E−21	BT1
l-Glutamic acid	1.30872	2.87953E−17	BT1
l-Histidine	1.09861	1.18916E−07	BT1
l-Isoleucine	1.09781	6.80041E−10	BT1
l-Leucine	1.26627	7.57847E−10	BT1
l-Proline	1.10174	3.35974E−23	BT1
l-Serine	1.14096	5.7145E−16	BT1
d-Phenylalanine	1.02392	9.58885E−21	BT2
l-Kynurenine	1.10698	2.25127E−06	BT2
*N*-Acetyl-l-glutamate	1.31966	6.89686E−08	BT2
*N*-Acetyl-l-leucine	1.20319	1.76547E−06	BT2

Among the compounds with significant variations, the substances related to biological control accounted to about 19.73%. There were a total of 4 antimicrobial substances identified in B with significantly high proportions and they included 2-aminoisobutyric acid, 5-aminolevulinic acid, dibutyl phthalate and gatifloxacin. Citric acid was found in abundance in T. Similarly, 5 antibacterial substances with significantly high yield were present in BT1 and included 4-hydroxybenzoic acid, apigenin, glycine betaine, malic acid and nicotinic acid, while another 5 antibacterial substances identified with significantly high yield in BT2 were indole-3-acetic acid, indole-3-carboxylic acid, phenacylamine, trans-3-coumaric acid and trans-cinnamic acid. Among these compounds, some inhibited fungus and some inhibited bacteria, while some displayed the additional functions of expelling insects or regulating plant growth (Table 3 and Fig. 4b). For example, the dibutyl phthalate abundantly present in B could potentially inhibit *Rhizoctonia solani* [18]. Likewise, citric acid, showing a high yield in T could effectively inhibit *Shigella* species [19]. Both glycine betaine and malic acid, present in high proportions in BT1 could effectively inhibit *Fusarium* sp. [20, 21]. Similarly, the indole-3-acetic acid with an elevated presence in BT2 could effectively inhibit *Fusarium solani f.* sp. *Eumartii* [22]. Some compounds identified were priorly proven for their antibacterial functions. For example, the 4-hydroxybenzoic acid with a significantly high yield in BT1 could effectively inhibit *Staphylococcus aureus* [23]. The trans-cinnamic acid with a significantly high yield in BT2 could inhibit *E. coli*, *Listeria* etc. [24]. For different types of plant diseases, the following targeted controls were conducted by selecting singly-cultured or co-culture models, and a certain specific compound could be developed through chemical separation method to prevent and control a specific disease. In addition, some compounds displayed other functions. For example, the 5-aminolevulinic acid with a high yield in B was capable of expelling insects and weeding. The indole-3-acetic acid with a high yield in BT2 could promote plant growth [25]. This establishes a foundation for insect-resistance and growth promotion for further applications in agriculture.

**Table 3:** Bio-control related metabolites with highly productive under only one culture condition

Compound	VIP	p-value	Culture condition	Function
2-Aminoisobutyric acid	1.36007	1.74E−07	B	*Candida albicans* [35]
5-Aminolevulinic acid	1.00584	0.002082456	B	Herbicide/insecticide [36]
Dibutyl phthalate	1.05246	0.007555153	B	*Rhizoctonia solani* [37]
Gatifloxacin	1.17378	1.15E−07	B	*Staphylococcus aureus* [38]
Citric acid	1.26357	1.65E−16	T	*Shigella* sp. [19, 39]
4-Hydroxybenzoic acid	1.32557	2.41E−12	BT1	*Staphylococcus aureus*, *Fusarium culmorum* [23]
Apigenin	1.35917	2.34E−18	BT1	*Alternaria tenuissima*, *Salmonella typhimurium* [40, 41]
Glycine betaine	1.13412	3.00E−20	BT1	*Fusarium verticillioides* [20]
Malic acid	1.3502	8.97E−23	BT1	*Aspergillus flavus*, *Fusarium oxysporum* [21]
Nicotinic acid	1.01745	1.01E−10	BT1	*Staphylococcus aureus*, *Aspergillus nige*r [42, 43]
Indole-3-acetic acid	1.34315	6.82E−11	BT2	*Fusarium solani* f. sp. *eumartii* [22]
Indole-3-carboxylic acid	1.31271	4.53E−06	BT2	*Plectosphaerella cucumerina* [44]
Phenacylamine	1.01991	3.66E−19	BT2	Bactria [45, 46]
Trans-3-coumaric acid	1.13656	0.000448228	BT2	*Escherichia coli*, *Staphylococcus aureus* [47]
Trans-cinnamic acid	1.03921	4.05E−18	BT2	*Fusicladium effusum*, *Listeria* [24, 48]

In the current study, many differential compounds were determined through methanol water extraction alone, aided with LC–MS/MS technology. It is possible to generate additional differential antibacterial compounds through other extraction and determination methods. By employing UPLC and NMR technologies, the unknown antibacterial compounds could also be isolated for identification and the modification types and new skeletons of novel compounds could be unraveled.

### The KEGG analysis of differential compounds in singly-cultured and co-cultured *B. amyloliquefaciens* ACCC11060 and/or *T. asperellum* GDFS1009 fermentation liquors

**Fig. 5:**
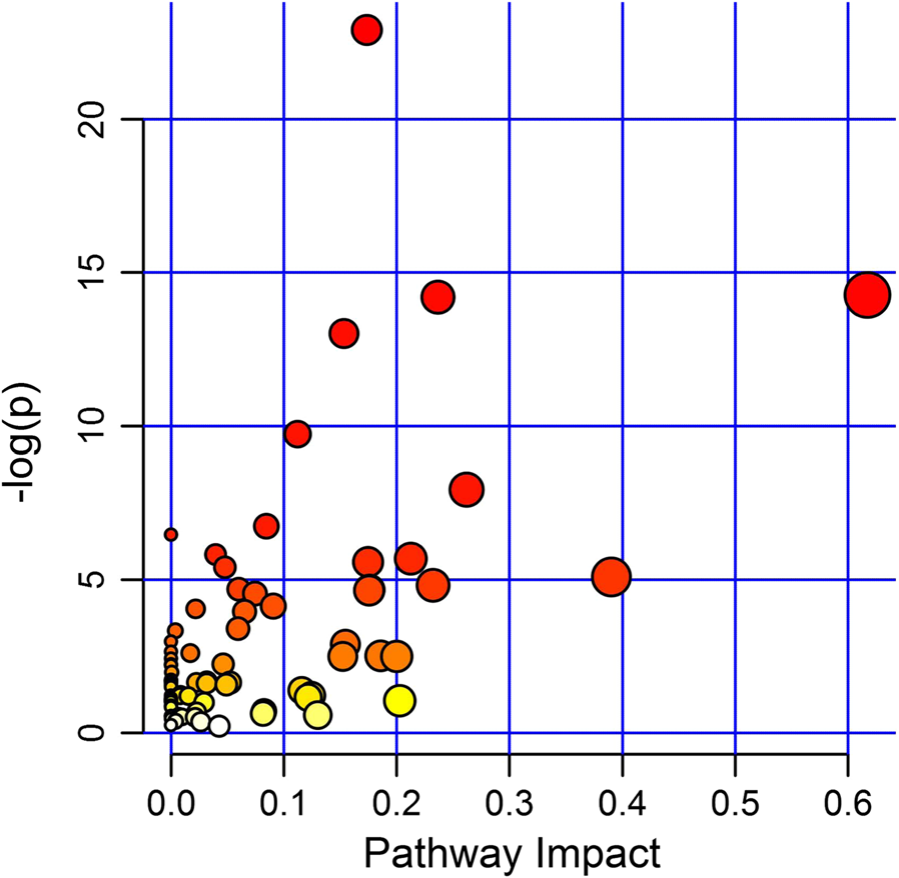
KEGG pathway analysis of the changed metabolites

A KEGG analysis was conducted with the 76 differential metabolites generated from the comprehensive comparison between B, T, BT1 and BT2. The integrative calculation of impact and −log (p) indicated that the 76 metabolites were involved in a total of 78 metabolic pathways (Fig. 5).

According to the rank of Impact numerical values, it was found that in 15 metabolic pathways with the most significant variations, 9 were related with amino acid metabolism such as the alanine, aspartate and glutamate metabolism, d-glutamine and d-glutamate metabolism, histidine metabolism, tryptophan metabolism, arginine and proline metabolism, lysine degradation, lysine biosynthesis, biosynthesis of amino acids, glycine, serine and threonine metabolism. The other five were taurine and hypotaurine metabolism, 2-oxocarboxylic acid metabolism, vitamin B6 metabolism, aminobenzoate degradation, tropane, piperidine and pyridine alkaloid biosynthesis, and citrate cycle (Table 4). Additionally, the metabolic pathways in which the differential compounds were involved included indole alkaloid biosynthesis, flavone and flavonol biosynthesis, flavonoid biosynthesis, biosynthesis of antibiotics etc. (data not shown).

**Table 4:** Significantly changed KEGG pathways

Pathway	Total	Hits	−LOG(p)	Impact
Alanine, aspartate and glutamate metabolism	28	7	14.271	0.61709
Taurine and hypotaurine metabolism	20	3	5.0872	0.39019
d-Glutamine and d-glutamate metabolism	8	3	7.9343	0.2619
2-Oxocarboxylic acid metabolism	108	12	14.199	0.23654
Vitamin B6 metabolism	22	3	4.8140	0.2322
Histidine metabolism	33	4	5.6843	0.21248
Aminobenzoate degradation	64	2	1.0488	0.20265
Tropane, piperidine and pyridine alkaloid biosynthesis	25	2	2.4945	0.20000
Tryptophan metabolism	53	3	2.5133	0.18578
Arginine and proline metabolism	68	5	4.6681	0.17587
Lysine degradation	44	4	4.6444	0.17555
Lysine biosynthesis	34	4	5.5736	0.17464
Biosynthesis of amino acids	127	17	22.916	0.17353
Citrate cycle (TCA cycle)	20	2	2.8901	0.15453
Glycine, serine and threonine metabolism	47	8	13.013	0.15325

Total, the total number of metabolites in the target metabolic pathway; Hits, the number of differential metabolites in the target metabolic pathway; −log (p): −log (p-value); impact, the greater the effect of metabolic pathways, the better

### Correlation analysis on the differential compounds in singly-cultured and co-cultured *B. amyloliquefaciens* ACCC11060 and/or *T. asperellum* GDFS1009 fermentation liquors

The importance of correlation analysis is to find the association between different compounds. In production, the yield of one compound can be increased by increasing the amount of another compound.

A correlation analysis on the 76 differential metabolites generated through the comprehensive comparison between B, T, BT1 and BT2 was conducted.

A positive correlation was present among d-aspartic acid, l-allothreonine, l-glutamic acid, l-isoleucine, l-proline and l-serine which had high yields in BT1. While the l-aspartic acid which displayed a high yield in T presented a positive correlation with them, l-lysine with high yield in B had no correlation with them at all. Further, l-kynurenine with a high yield in BT2 posed a negative correlation with them. In addition, the citric acid with a high yield in T displayed a positive correlation with d-aspartic acid, l-allothreonine, l-glutamic acid, l-isoleucine, l-proline and l-serine.

**Fig. 6:**
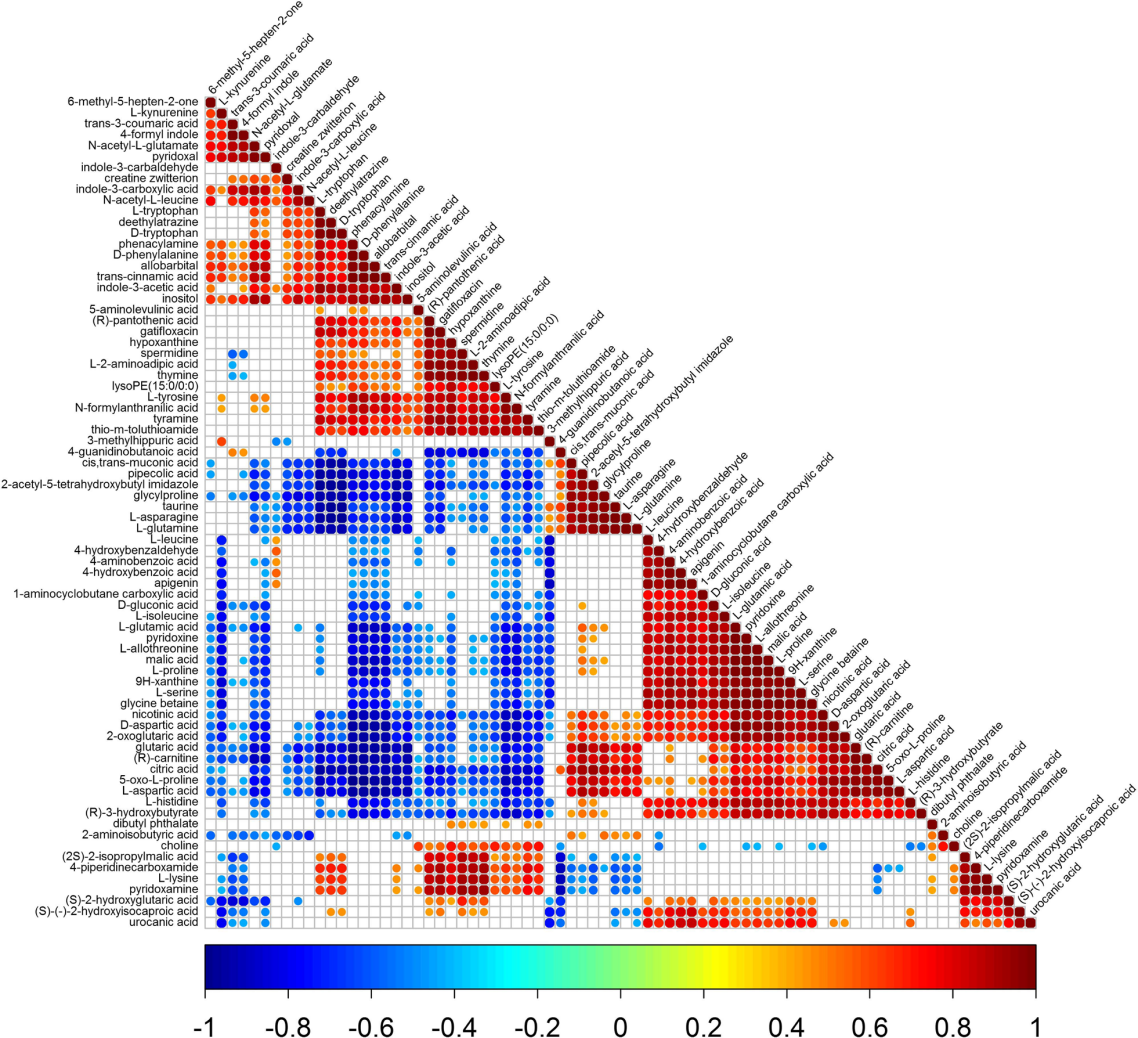
Correlation analysis of the changed metabolites. Dark red represents a significant positive correlation. Dark blue represents a significant negative correlation. White represents no correlation

The 5-aminolevulinic acid which exhibited a high yield in B had no correlation with 2-acetyl-5-tetrahydroxybutyl imidazole which had a high yield in T. Similarly, the apigenin with a high yield in BT1 displayed no correlation with indole-3-acetic acid which had a high yield in BT2 (Fig. 6).

This information provides a technical guidance for generating a combination of compounds for use in agriculture or industry. During large scale production of compounds, the same observation may be considered for the purpose of increasing the generation of amino acids by increasing the ratio of citric acid in the medium.

## Conclusions

When an inoculation proportion between *B. amyloliquefaciens* ACCC11060 and *T. asperellum* GDFS1009 is 1.9:1, the antimicrobial effects of the resulting co-cultured fermentation liquor is significantly higher than those of single cultivations. The syntheses of some antimicrobial substances contributed to the observed increase. In addition, when the inoculation proportion between *B. amyloliquefaciens* ACCC11060 and *T. asperellum* GDFS1009 is 1:1, the co-cultivation would enhance the production of specific amino acids. This could be further explored for a large scale production of amino acids or at least serve as a theoretical basis for the generation of certain rare amino acids.

## Methods

### Strains

The biocontrol bacterium *B. amyloliquefaciens* ACCC11060 was purchased from Agricultural Culture Collection of China (ACCC NO. 11060), Beijing, China. The biocontrol fungus *T. asperellum* GDFS1009 was preserved in China General Microbiological Culture Collection Center (CGMCC NO. 9512), Beijing, China. *B. cinerea* on tomato was used as a target pathogen for the examination of biocontrol effects.

### The co-cultured combinations

The liquid BP broth (Beef extract 0.3% and peptone 0.5%) was used in this study for both pure and co-cultivation.

*Trichoderma asperellum* GDFS1009 was incubated at a constant temperature of 28 °C in PAD plates for 3 days, following which the spores were washed with sterile water, filtered through degreasing cotton, inoculated into BP broth to a final concentration of 10^6^ cfu/mL, and cultured with shaking at 180 rpm overnight at 28 °C, until the OD_600_ reached 1.0. *B. amyloliquefaciens* ACCC11060 was incubated in LB culture dishes for 2 days, following which the cells were washed with sterile water, inoculated into BP broth, and cultured with shaking at 180 rpm overnight at 28 °C until the OD_600_ reached 1.0.

1 mL of *B. amyloliquefaciens* ACCC11060 was singly inoculated into 100 mL BP broth, fermented at 180 rpm and 28 °C for 3 days, and the resulting fermentation liquor was named as B. Similarly, 1 mL of *T. asperellum* GDFS1009 was singly inoculated into 100 mL BP broth, fermented at 180 rpm and 28 °C for 3 days, and the resulting fermentation liquor was labelled as T.

1 mL each of *B. amyloliquefaciens* ACCC11060 and *T. asperellum* GDFS1009 in equal proportions were inoculated into 100 mL BP broth, co-cultured at 180 rpm and 28 °C for 3 days, and the resulting fermentation liquor was named as BT1. Lastly, 1.9 mL of *B. amyloliquefaciens* ACCC11060 and 1 mL of *T. asperellum* GDFS1009 were inoculated into 100 mL BP broth, co-cultured at 180 rpm and 28 °C for 3 days, and the resulting fermentation liquor was labeled as BT2.

Apart from these, many other combination models were attempted with different inoculation ratios of *Trichoderma* sp. and *Bacillus* sp. As they were not typical (Neither natural trial option nor best inhibition on *B. cinerea*), we didn’t descript it for details. Each group of experiments had three repeats.

### Antibacterial effects

*Botrytis cinerea* was incubated at a constant temperature of 25 °C in PAD culture dishes for 5 days, and the fungus cake was passed through a 7 mm hole punch. 10 mL each of the above multiple groups of co-cultured and singly-cultured fermentation liquors, along with 10 mL of blank BP broth serving as control, were filtered through Millipore filters of pore size 0.22 µm and added into 40 mL of PDA broth at a temperature of 55 °C, following which the contents were mixed well and poured into plates. The fungus cakes of *B. cinerea* were then inversely placed in the middle of each plate and incubated at a constant temperature of 25 °C for 3 days. The colony diameters were determined by applying the ‘crossing method’ and photographed. Each group of experiments had three repeats.

### Preparation of samples for LC–MS/MS

50 mL each of the fermentation liquors B, T, BT1 and BT2, along with 50 mL of blank BP broth serving as control, were filtered through Millipore filters of pore size 0.22 µm and snap-frozen with liquid nitrogen, following which they were transported on dry ice to Suzhou BioNovoGene Metabolomics Platform.

All the samples were thawed at 4 °C and mixed uniformly. 200 µL of each sample was taken in a 1.5 mL microcentrifuge tube, to which 800 µL of methyl alcohol was added and vibrated for 60 s to mix the contents uniformly, following which the tubes were centrifuged at 12,000 rpm at 4 °C for 10 min. The supernatants were transferred to a new 1.5 mL microcentrifuge tube, concentrated and dried by vacuum and re-dissolved in 300 µL of 80% methyl alcohol. Next, the supernatants were filtered through a 0.22 µm membrane to obtain the samples for determination. 20 µL of each sample was kept aside for QC, while the remaining was analyzed through LC–MS [26, 27].

For the chromatographic study, Waters ACQUITY UPLC instrument was used with ACQUITY UPLC^®^ BEH C18 1.7 µm (2.1 × 100 mm) chromatographic column. The temperature of automatic sampler was set to 4 °C with a flow velocity of 0.25 mL/min. The column temperature was 40 °C. 10 µL of sample was introduced for gradient elution, and the mobile phase was 0.1% formic acid water (A)—0.1% formic acid acetonitrile (B). The gradient elution program was as follows: 0–1 min, 2% B; 1–9.5 min, 2–50% B; 9.5–14 min, 50–98% B; 14–15 min, 98% B; 15–15.5 min, 98–2% B; 15.5–17 min, 2% [28].

For the mass spectrometry study, the Thermo LTQ Orbitrap XL instrument was used with electrospray ionization (ESI) and cation–anion ionization mode. The voltages for positive and negative ion sprays were 4.80 kV and 4.50 kV respectively. The sheath gas was 45arb and auxiliary gas was 15arb. The capillary temperature was 325 °C, and the capillary voltage was 35 V/− 15 V. The voltage of tube lens was 50 V/− 50 V and the full scan was conducted with the resolution of 60,000, with the scan scope being 89–1000. CID was applied for secondary dissociation with a fragmentor voltage of 30 eV. Simultaneously, dynamic exclusion (with the repeat count of 2) was applied to eliminate the unnecessary MS/MS information, with the time for dynamic exclusion set as 15 s [29].

### Analysis of metabolome data

The data pre-processing included the following: (a) the original data obtained was converted into mzXML format (xcms input file format) using Proteowizard software (v3.0.8789) [30]. (b) The XCMS software package of R (v3.3.2) was applied for peaks identification, filtration and alignment. The major parameters included bw = 5, ppm = 15, peakwidth = c (10,120), mzwid = 0.015, mzdiff = 0.01 and method = “centWave” [31]. (c) The data matrix containing the information of mass to charge ratio (m/z), retention time and peak area (intensity) was obtained. The positive and negative ion models gathered 3680 and 3566 precursor molecules respectively. The data were collected and the following analyses were conducted. (d) For the comparison between data of different orders, the batch normalization of peak area was conducted.

Few multivariate statistical analyses including principal component analysis (PCA) and Orthogonal Projections to Latent Structures Discriminant Analysis (OPLS-DA) were conducted to reveal the differences in the metabolic compositions between different comparison groups [32].

The LIGAND database of KEGG included the information related to chemical substances, enzyme molecules and reactions, while the metabolic pathways which the chemical compounds with specific differences were involved in were analyzed through KEGG [33].

The correlations between the metabolites were analyzed by calculating the Pearson or Spearman Rank correlation coefficient between any two metabolites. When the linear relation between two metabolites increased, the correlation coefficient trended toward 1 or − 1 i.e., it trended towards 1 during a positive correlation and − 1 during a negative correlation, while the calculation method was the cor() function in R (v3.1.3). The correlation analyses of metabolites and statistical test for significance were conducted simultaneously and the statistical test method was the cor.test() function in R language package. In addition, the false positive check on p-value was conducted and FDR p-value ≤ 0.05 was used as the significant correlation [34].

## Additional files

**Additional file 1.** Categories of all annotated compounds based on LC–MS/MS.

**Additional file 2.** Details of all annotated compounds based on LC–MS/MS.
