# Supplementary figures and images for: Co-culture of Bacillus amyloliquefaciens ACCC11060 and Trichoderma asperellum GDFS1009 enhanced pathogen-inhibition and amino acid yield
Source: Microb Cell Fact. 2018 Oct 3;17:155. doi: 10.1186/s12934-018-1004-x (PMC6171294; doi:10.1186/s12934-018-1004-x)

**Additional files**

**Additional file 1 Categories of all annotated compounds based on LC-MS/MS**

**
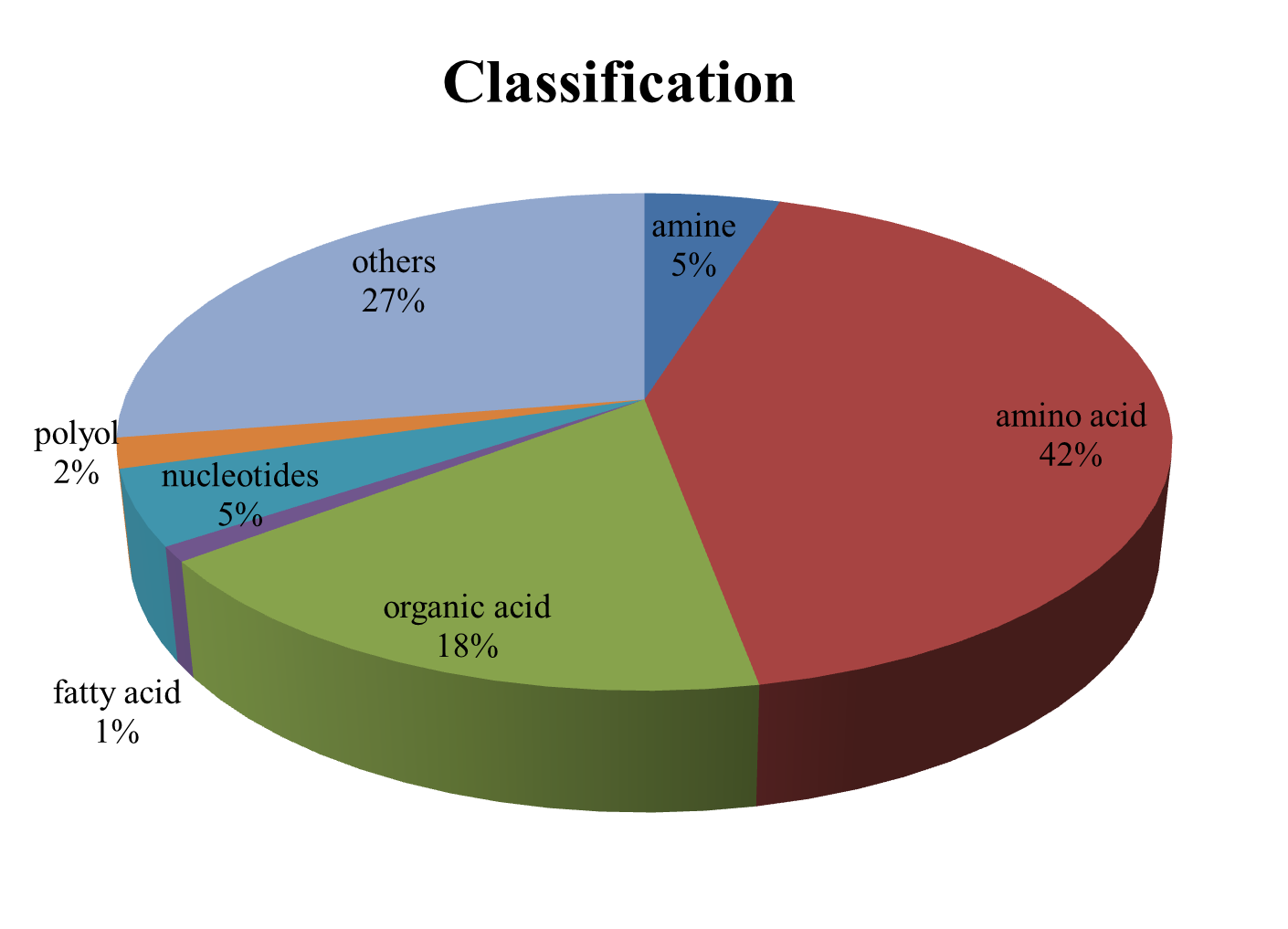
**

Supplement: Supplementary file 1 — Additional file 1. Categories of all annotated compounds based on LC–MS/MS. [file 12934_2018_1004_MOESM1_ESM.docx]
