# Supplementary material for: Co-culture of Bacillus amyloliquefaciens ACCC11060 and Trichoderma asperellum GDFS1009 enhanced pathogen-inhibition and amino acid yield
Source: Microb Cell Fact. 2018 Oct 3;17:155. doi: 10.1186/s12934-018-1004-x (PMC6171294; doi:10.1186/s12934-018-1004-x)
Supplement: Supplementary file 2 — Additional file 2. Details of all annotated compounds based on LC–MS/MS. [file 12934_2018_1004_MOESM2_ESM.docx]

**Additional files**

**Additional file 2 Details of all annotated compounds based on LC-MS/MS**

| **Name** | **Classification** | **Precursor type** | **B** **(AV)** | **T** **(AV)** | **BT1** **(AV)** | **BT2** **(AV)** |
| --- | --- | --- | --- | --- | --- | --- |
| 5-oxo-L-proline | amino acid | [M-H]- | 444587.2 | 87369551 | 73625953 | 3464518 |
| 2-aminoisobutyric acid | amino acid | [M+H]+ | 46240669 | 44175024 | 20233376 | 7977414 |
| choline | others | [M+H]+ | 57497907 | 17330646 | 5157362 | 1127378 |
| (R)-3-hydroxybutyrate | others | [M+H]+ | 184329.1 | 3607509 | 5131855 | 1285184 |
| L-serine | amino acid | [M+H]+ | 77631.91 | 2296160 | 8352027 | 124049.6 |
| trans-4-hydroxy-L-proline | amino acid | [M-H]- | 6478470 | 4568147 | 4201596 | 3423597 |
| creatine zwitterion | amino acid | [M-H]- | 3789371 | 2989458 | 4173510 | 4588854 |
| L-leucine | amino acid | [M-H]- | 148082.8 | 150302.7 | 2130046 | 133509.2 |
| glutaric acid | organic acid | [M-H]- | 370451.3 | 1814833 | 1364835 | 235035.4 |
| L-asparagine | amino acid | [M-H]- | 104045.2 | 1843509 | 323833.7 | 88087.35 |
| (S)-(-)-2-Hydroxyisocaproic acid | fatty acid | [M-H]- | 36905601 | 10709419 | 59613528 | 15928157 |
| D-aspartic acid | amino acid | [M-H]- | 426748.7 | 18674173 | 24988463 | 272326 |
| malic acid | organic acid | [M-H]- | 947992.2 | 96364581 | 1.86E+08 | 861905.2 |
| creatinine | amino acid | [M+H]+ | 1.6E+08 | 1.36E+08 | 1.28E+08 | 1.72E+08 |
| hypoxanthine | nucleotides | [M-H]- | 17380655 | 179271.6 | 509804.5 | 5183626 |
| 4-hydroxybenzoic acid | organic acid | [M-H]- | 98815.93 | 44651.3 | 1561655 | 151591 |
| urocanic acid | organic acid | [M-H]- | 395499.4 | 138465.9 | 937996.4 | 238755.3 |
| name | classification | [M-H]- | 236649.9 | 1791457 | 452949.6 | 71715.57 |
| 6-oxo-L-proline | amino acid | [M+H]+ | 4368410 | 2.05E+08 | 4.28E+08 | 3611452 |
| 3-aminoisobutyric acid | amino acid | [M+H]+ | 894249.9 | 7413451 | 35451059 | 6137721 |
| choline | others | [M-H]- | 1574552 | 82514.45 | 46241.08 | 2201472 |
| (R)-4-hydroxybutyrate | others | [M-H]- | 718757.7 | 7213190 | 9230599 | 791438.1 |
| L-serine | amino acid | [M+H]+ | 2402270 | 2270887 | 1123476 | 2191836 |
| trans-5-hydroxy-L-proline | amino acid | [M-H]- | 497333.5 | 4682915 | 526520.3 | 88106.63 |
| creatine zwitterion | amino acid | [M+H]+ | 30208758 | 2.58E+08 | 7E+08 | 33961272 |
| L-leucine | amino acid | [M-H]- | 1976389 | 11066344 | 19211589 | 956636.3 |
| glutaric acid | organic acid | [M+H]+ | 439373.5 | 11478835 | 26912152 | 798200.6 |
| L-asparagine | amino acid | [M-H]- | 1551394 | 905790.5 | 1749721 | 500952.4 |
| (S)-(-)-3-Hydroxyisocaproic acid | fatty acid | [M+H]+ | 125850 | 358944 | 1909145 | 633943.7 |
| D-aspartic acid | amino acid | [M+H]+ | 61260179 | 90126135 | 99500565 | 65662103 |
| malic acid | organic acid | [M+H]+ | 158058.2 | 4703658 | 317747 | 187955.6 |
| creatinine | amino acid | [M+H]+ | 15073947 | 445283.3 | 4992188 | 5715711 |
| hypoxanthine | nucleotides | [M+H]+ | 16828685 | 16527488 | 16419633 | 21833285 |
| 5-hydroxybenzoic acid | organic acid | [M-H]- | 1666587 | 6109408 | 15993642 | 739216.7 |
| urocanic acid | organic acid | [M-H]- | 9320567 | 1210319 | 747537.6 | 9054760 |
| name | classification | [M-H]- | 302287.8 | 35996.35 | 54707.83 | 314710.5 |
| 7-oxo-L-proline | amino acid | [M+H]+ | 4497556 | 868843 | 3056531 | 1850075 |
| 4-aminoisobutyric acid | amino acid | [M-H]- | 248902.6 | 1032254 | 1561018 | 489806.8 |
| choline | others | [M-H]- | 243532.1 | 33270.04 | 247163.2 | 485083.8 |
| (R)-5-hydroxybutyrate | others | [M-H]- | 541103.8 | 24580.06 | 180422.8 | 230099.2 |
| L-serine | amino acid | [M-H]- | 328770.6 | 1516377 | 938411.9 | 15343511 |
| trans-6-hydroxy-L-proline | amino acid | [M+H]+ | 2286264 | 24735554 | 10668827 | 2209388 |
| creatine zwitterion | amino acid | [M-H]- | 552328.1 | 59521.8 | 89842.2 | 365728.3 |
| L-leucine | amino acid | [M-H]- | 77908160 | 8860998 | 9932109 | 73194413 |
| glutaric acid | organic acid | [M+H]+ | 6060566 | 2987558 | 2149073 | 3208557 |
| L-asparagine | amino acid | [M+H]+ | 4635171 | 30754224 | 1.32E+08 | 2810816 |
| (S)-(-)-4-Hydroxyisocaproic acid | fatty acid | [M-H]- | 1318589 | 61947.44 | 64151.62 | 610096.9 |
| D-aspartic acid | amino acid | [M+H]+ | 1626245 | 18544203 | 14299281 | 98201.27 |
| malic acid | organic acid | [M+H]+ | 488482.6 | 2428316 | 3355002 | 667595.1 |
| creatinine | amino acid | [M+H]+ | 18922480 | 1577298 | 3476053 | 19615790 |
| hypoxanthine | nucleotides | [M+H]+ | 364891.3 | 1121041 | 8753899 | 212016.4 |
| 6-hydroxybenzoic acid | organic acid | [M+H]+ | 3907620 | 59049.51 | 997086 | 1746036 |
| urocanic acid | organic acid | [M-H]- | 3081311 | 208700.8 | 296923.7 | 3466196 |
| name | classification | [M-H]- | 5601887 | 585732.3 | 3593170 | 6504908 |
| 8-oxo-L-proline | amino acid | [M+H]+ | 289625.4 | 294321.3 | 318697.2 | 4849197 |
| 5-aminoisobutyric acid | amino acid | [M-H]- | 2725598 | 789612.2 | 2201295 | 1153298 |
| choline | others | [M+H]+ | 4850178 | 4625569 | 5956656 | 5742316 |
| (R)-6-hydroxybutyrate | others | [M+H]+ | 1191238 | 6886307 | 3016392 | 5102076 |
| L-serine | amino acid | [M-H]- | 6063265 | 463793.5 | 3341890 | 8435940 |
| spermidine | amine | [M+H]+ | 35671532 | 9526926 | 17210571 | 14475250 |
| L-tyrosine | amino acid | [M-H]- | 79241786 | 6691982 | 9777199 | 49198520 |
| acetylcholine chloride | others | [M-H]- | 5853145 | 8341266 | 9260552 | 8961333 |
| L-lysine | amino acid | [M+H]+ | 3.96E+08 | 46774288 | 2.9E+08 | 1.29E+08 |
| galactitol | polyol | [M-H]- | 2544803 | 3279592 | 2172329 | 4513011 |
| trans-cinnamic acid | organic acid | [M+H]+ | 5546802 | 762234.7 | 1232100 | 6711958 |
| L-methionine | amino acid | [M+H]+ | 9.78E+08 | 73879457 | 2.83E+08 | 1.01E+09 |
| thio-m-Toluthioamide | amine | [M+H]+ | 79708807 | 241498.6 | 1198667 | 28939345 |
| 4-hydroxyphenylacetic acid | organic acid | [M+H]+ | 2308943 | 3176344 | 1529052 | 1880556 |
| N-acetyl-L-glutamate | amino acid | [M-H]- | 403219.8 | 12286.91 | 120711.8 | 875116.6 |
| citric acid | organic acid | [M-H]- | 1976225 | 30628107 | 19850303 | 4753674 |
| 3-Methylhippuric acid | amino acid | [M-H]- | 667755.3 | 828547.2 | 268852.3 | 692557.1 |
| D-gluconic acid | organic acid | [M-H]- | 477675.3 | 626564 | 887612.1 | 491967.5 |
| (R)-carnitine | amino acid | [M+H]+ | 25558429 | 1.29E+08 | 1.09E+08 | 20988652 |
| trans-4-coumaric acid | organic acid | [M+H]+ | 63345784 | 6383710 | 19798217 | 62211505 |
| trans-2-coumaric acid | organic acid | [M+H]+ | 7088842 | 1210961 | 1652084 | 7977757 |
| L-tryptophan | amino acid | [M-H]- | 25653453 | 4467484 | 16903359 | 22854905 |
| N-formylanthranilic acid | amino acid | [M+H]+ | 10502782 | 2971867 | 1996247 | 7776868 |
| D-phenylalanine | amino acid | [M+H]+ | 5.01E+09 | 1.04E+09 | 1.32E+09 | 5.22E+09 |
| allobarbital | others | [M-H]- | 4568657 | 335325.9 | 414351.9 | 6017051 |
| pyridoxal | others | [M+H]+ | 3480244 | 374459.9 | 33303.85 | 7848816 |
| pyridoxamine | others | [M+H]+ | 78133131 | 12290112 | 53648154 | 26824025 |
| pyridoxine | others | [M+H]+ | 29512.1 | 1156895 | 2428515 | 71539.25 |
| (R)-pantothenic acid | others | [M-H]- | 18572545 | 475108.5 | 7662871 | 10985150 |
| Glycylproline | amino acid | [M+H]+ | 2373137 | 9190764 | 3451510 | 852231 |
| N-acetyl-L-leucine | amino acid | [M+H]+ | 1032538 | 409858.1 | 1319688 | 2072498 |
| L-cystine | amino acid | [M-H]- | 290040.8 | 367096 | 375595.5 | 294006.3 |
| N-acetyltryptophan | amino acid | [M-H]- | 159700.5 | 43702.63 | 34133.94 | 171872.8 |
| deethylatrazine | others | [M+H]+ | 1.62E+08 | 44543201 | 1.3E+08 | 1.48E+08 |
| adenosine | nucleotides | [M-H]- | 382609.7 | 257756.7 | 241178.3 | 262262.3 |
| apigenin | others | [M-H]- | 371302.2 | 58754.02 | 4836928 | 227425.7 |
| D-tryptophan | amino acid | [M+H]+ | 1.26E+09 | 4.18E+08 | 1.05E+09 | 1.18E+09 |
| N-acetyl-L-phenylalanine | amino acid | [M+H]+ | 335286 | 395868 | 487645.7 | 470103.8 |
| L-kynurenine | amino acid | [M+H]+ | 8513416 | 8209400 | 5275898 | 10093710 |
| 2-Acetyl-5-tetrahydroxybutyl Imidazole | others | [M+H]+ | 257998.2 | 2951033 | 1067001 | 395955.8 |
| N-acetyl-beta-neuraminic acid | amino acid | [M-H]- | 1307783 | 990427.5 | 881958.1 | 1453720 |
| daidzein | others | [M+H]+ | 700360.9 | 919207.8 | 915831.9 | 946387.1 |
| dibutyl phthalate | others | [M+H]+ | 4483307 | 3651396 | 3566149 | 3172223 |
| 13,14-dihydro-15-keto-tetranor PGE2 | others | [M+H]+ | 9479663 | 3860414 | 5433010 | 10667663 |
| Clomipramine | others | [M+H]+ | 2863055 | 625901.5 | 1284587 | 2500224 |
| Tris(1-chloro-2-propyl)phosphate | others | [M+H]+ | 493775 | 774107.2 | 632116.5 | 373986 |
| gatifloxacin | others | [M+H]+ | 18663578 | 6832458 | 12292046 | 12837375 |
| LysoPE(15:0/0:0) | others | [M+H]+ | 2957956 | 658681 | 243286.7 | 1330109 |

AV means average value.
